# Gastric Cancer Stem Cells: A Glimpse on Metabolic Reprogramming

**DOI:** 10.3389/fonc.2021.698394

**Published:** 2021-06-16

**Authors:** Martina Addeo, Giuseppina Di Paola, Henu Kumar Verma, Simona Laurino, Sabino Russi, Pietro Zoppoli, Geppino Falco, Pellegrino Mazzone

**Affiliations:** ^1^ Istituto di Ricerche Genetiche Gaetano Salvatore Biogem Scarl, Ariano Irpino, Italy; ^2^ Department of Biology, University of Naples Federico II, Naples, Italy; ^3^ IEOS-CNR, Institute of Experimental Endocrinology and Oncology “G. Salvatore” – National Research Council, Naples, Italy; ^4^ Laboratory of Pre-Clinical and Translational Research, Istituto di Ricovero e Cura a Carattere Scientifico (IRCCS)-centro di riferimento oncologico della basilicata (CROB), Referral Cancer Center of Basilicata, Rionero in Vulture, Italy

**Keywords:** gastric cancer, cancer stem cell (CSC), metabolism, reprogramming, therapy

## Abstract

Gastric cancer (GC) is one of the most widespread causes of cancer-related death worldwide. Recently, emerging implied that gastric cancer stem cells (GCSCs) play an important role in the initiation and progression of GC. This subpopulation comprises cells with several features, such as self-renewal capability, high proliferating rate, and ability to modify their metabolic program, which allow them to resist current anticancer therapies. Metabolic pathway intermediates play a pivotal role in regulating cell differentiation both in tumorigenesis and during normal development. Thus, the dysregulation of both anabolic and catabolic pathways constitutes a significant opportunity to target GCSCs in order to eradicate the tumor progression. In this review, we discuss the current knowledge about metabolic phenotype that supports GCSC proliferation and we overview the compounds that selectively target metabolic intermediates of CSCs that can be used as a strategy in cancer therapy.

## Introduction

Gastric cancer (GC) is recognized as the fifth most common cancer in the world (5.7% of all cancers) with a higher prevalence in males compared to females, and it is the fourth leading cause of cancer death (1). Lifestyle variations, especially in dietary habits such as high salt intake, iron depletion and alcohol consumption, along with genetic background, have led to a discrepancy in GC incidence in different regions of the world with Europeans and Latinos less affected than Asians (2–4). The high mortality rate could be accounted by the absence of early-stage symptomatology, by the lack of early diagnosis and poorly effective treatments (5). Gastric cancer is classified as a multifactorial disease that results from a combination of specific genetic alterations such as gene mutations, somatic copy number alterations (sCNAs), epigenetic changes (6–8), and environmental factors (9). The latter has a critical role in the GC onset, with major risk factors being *Helicobacter pylori *infection (10). At the present, *H. pylori* infection is a strong risk factor for the adenocarcinoma that arises within the stomach (11), and it was classified as a class I carcinogen by the International Agency for Research on Cancer (12). *H. pylori* colonizes the gastric mucosa, where it expresses an array of proteins that lead to persistent inflammation (13, 14). All these factors together promote cancer stem cells insurgence even if it is unclear whether this mechanism is due to a somatic cancer cell that acquires stemness feature, or to a normal stem cell which acquires cancer properties.

### Hallmarks of Cancer Stem Cells (CSCs)

Cancer stem cells (CSCs) represent a small subpopulation within the tumor that is involved in the initiation and progression of carcinomas (15–17). According to the CSCs theory, the tumor bulk is composed of a plethora of heterogeneous and differentiated cancer cells which is fueled by a rare population of CSCs characterized by self-renewal and differentiation capabilities (18, 19).

The molecular features displayed by CSCs are not universal and many of the genes, discovered as markers of these cells, were first identified in embryonic stem cells (ESCs). The core regulatory network for embryonic stem cell maintenance and self-renewal *OCT4, SOX2, KLF4, NANOG*, and *SALL4* are abnormally expressed in human tumor samples suggesting the presence of cancer stem cells (20). The overexpression of these pluripotency genes in gastric cancer tumor tissues *versus* the paired adjacent normal tissues positively correlate with tumor size, tumor grade, TNM stage, and shortened overall survival time (21). *Zscan4*, a transcription factor (TF) firstly identified as exclusive of murine 2-cell embryos (22) and murine ESCs (mESCs) (23), is also been associated with stem cell phenotype in human head and neck squamous cell carcinoma (HNSCC) (24). ZSCAN4 is indeed enriched in HNSCC cells which are able to form tumorspheres, and its overexpression is associated with elevated histone 3 hyperacetylation at NANOG and OCT4 promoters. The TF *c-MYC* is one of the most studied oncogenes, and originally part of Yamanaka cocktail together with *OCT4, SOX2* and *KLF4* (OSKM), to reprogram somatic cell to a pluripotent cell (25). The reactivation of MYC in mammary epithelial cells is able to downregulate lineage specific TFs to reprogram the cell to a stem cell-like state favoring tumor initiation and progression (26). As the CSCs could be considered normal stem cells which have lost control over regulation mechanisms, they can also take advantage of other protective mechanisms typical of stem/progenitor cells. The adenosine triphosphate–binding cassette (ABC) transporters are efflux pumps expressed at high level on progenitor cells membrane and are responsible for the protection of the stem cell population from toxic molecules (27). Although the expression of ABC transporters strongly fosters multidrug resistance (MDR) (28), the capacity of CSCs to resist chemotherapeutic treatment is a multifactorial feature achieved by a highly efficient DNA repair machinery that is employed to overcome the DNA damage induced by therapeutic treatment (28) as well as increasing the autophagic process to obtain nutrients necessary to support cell survival (28, 29). Also, upon drug treatment, other molecular pathways may take action in sustaining the survival of CSCs. VEGF/VEGFR-1(Flt) autocrine signaling is activated in a subpopulation of highly tumorigenic cells in response to cisplatin treatment (CDDP) and is characterized by the expression of the pluripotency genes *OCT4, NANOG* and *BMI1* (30). The drug exposure can therefore enhance the tumorigenic potential of the CSCs hence revealing the need to use combined therapies in order to target different molecular pathways and reduce the chance of cancer relapse.

### Cancer Stem Cells Metabolism

**Figure 1:**
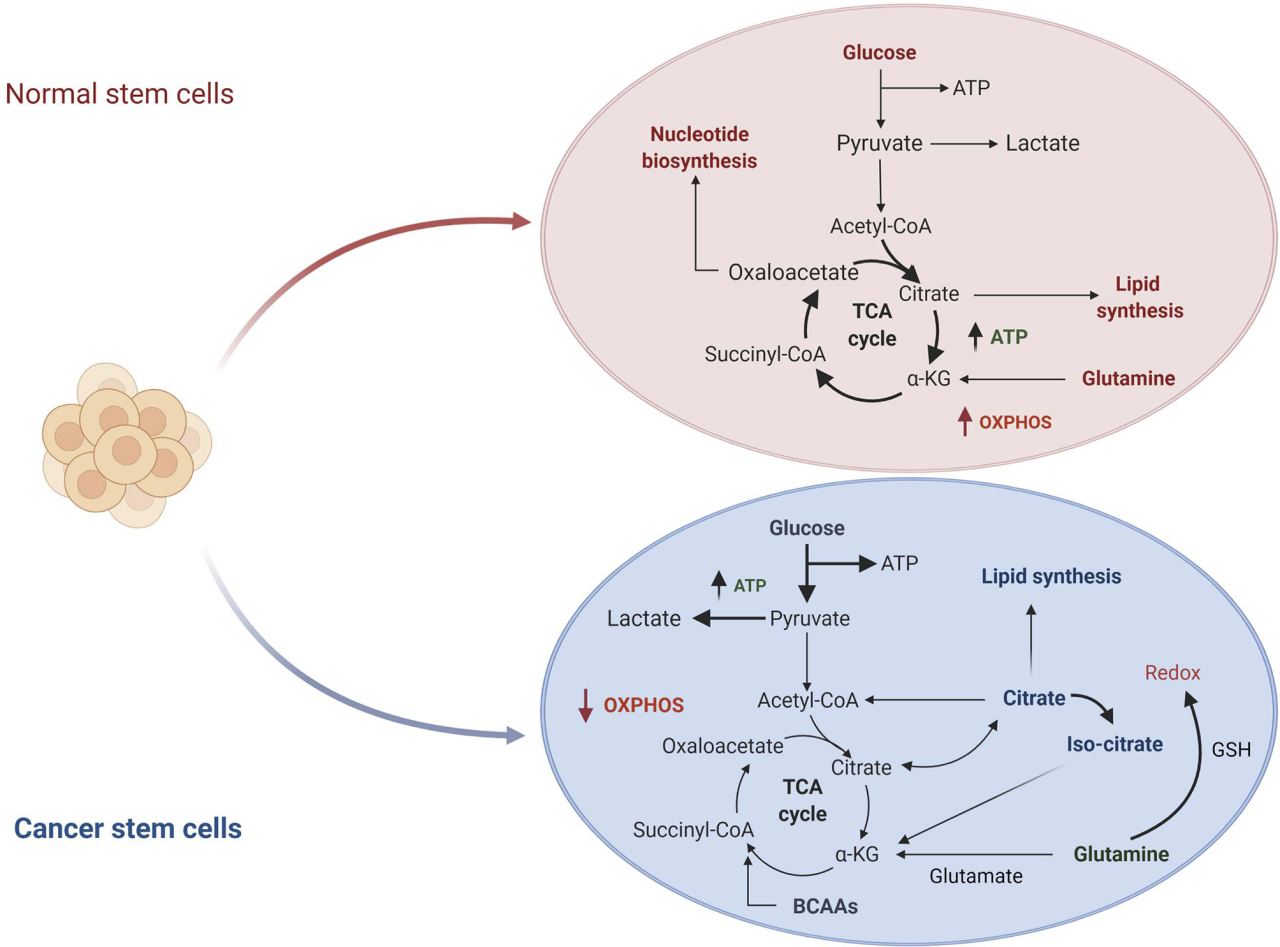
Comparison of normal stem cells *versus* cancer stem cells metabolism. In normal cells, glucose is converted to pyruvate and then oxidized to Acetyl-CoA in the mitochondrial TCA cycle. Other substrates, including lipids and nucleotides, are produced from these catabolic reactions. The majority of ATP is produced by oxidative phosphorylation (OXPHOS) (not shown in the figure). In cancer cells, glucose uptake and glycolysis are strongly increased. Part of pyruvate is converted to lactate, while the remaining is converted to Acetyl-CoA to enter in the TCA cycle where it is converted to citrate. The produced citrate is secreted from the mitochondria for fatty acid synthesis that are necessary for structural maintenance of the membrane. TCA, tricarboxylic acid cycle; *α*KG, *α*-KetoGlutarate; GSH, glutathione; BCAAs, branched-chain amino acids. Figure modified from De Berardinis et al. (34).

The metabolic hallmarks of the CSCs have been the subject of intensive investigation in different types of tumors (31, 32). A common feature of CSCs is the reprogramming of cellular metabolism with glycolysis preferred over oxidative phosphorylation (OXPHOS) as the primary source of ATP molecules even in presence of oxygen. This metabolic switch, known as the “Warburg effect” or aerobic glycolysis, provides ready-to-use energy that is essential to meet the need of high energy demand associated with a high proliferative state (33). Although this mechanism is energetically unfavorable, as the amount of ATP generated by glycolysis is lower than the quantity deriving from OXPHOS, CSCs overcome the energy limitation enhancing glucose uptake and upregulating some intermediates of the glycolytic pathway. On the other hand, in normal cells or quiescent somatic cells, mitochondria produce the primary energy through the tricarboxylic acid cycle (TCA) deriving from glucose *via* glycolysis, or fatty acid *via* β-oxidation associated with OXPHOS (**Figure 1**) (35). All these metabolic changes allow the CSCs adaptation to the tumor microenvironment leading to tumor progression, metastases formation and chemo-resistance (36).

In T-cell leukemia the activation of MYC leads to HIF2α induction that is facilitated by the pluripotency factors NANOG and SOX2. This results in a change of redox balance of CSCs as HIF2α negatively regulates p53 activation and positively regulates glutathione (GSH) production (37). Furthermore, several studies reported that an aberrant activation of WNT signaling plays an important role in the metabolic switch from an oxidative metabolism to aerobic glycolysis supporting cancer stem cells. This shift could be partly explained by the production of reactive oxygen species (ROS) as they are capable of altering the self-renewal capacity of cancer stem cells (38). As a matter of fact, WNT has been shown to regulate pyruvate dehydrogenase kinase, PDK1, by phosphorylating and inactivating the pyruvate dehydrogenase (PDH) enzyme complex, responsible for the conversion of pyruvate into Acetyl-CoA (39). A decreased entry of Acetyl-CoA into TCA and oxidative phosphorylation leads to a reduced mitochondrial respiration and, therefore, to lower ROS levels. The role of WNT in supporting the stemness of CSCs was further confirmed by the use of a WNT antagonist, secreted frizzled-related protein 4 (sFRP4). This protein, through its binding to WNT receptor, is able to reduce the CSCs viability even in the presence of a variable concentration of glucose (40).

Moreover, Notch1 signaling plays a role in the regulation of metabolism in CSCs. In particular, upon binding to Jagged1 ligand, Notch1 activates a downstream cascade that, through the interaction with PTEN-induced kinase 1 (PINK1), results in mTORC2/AKT activation (41). In CSCs, mTORC2 regulates different mechanisms through the AKT activation which, in turn, phosphorylates SOX2 and OCT4 and, therefore, it positively contributes to the stemness maintenance. Furthermore, mTORC2 activation is also implied in metabolic regulation as it is involved in the repression of the FoxO3, a transcriptional factor that is responsible for the inhibition of the glycolytic pathway (42).

Besides, Hedgehog signaling (Hh) plays an important role in CSCs sustenance. A recent study describes a role of this pathway in the activation of SOX2, NANOG and OCT4 stemness genes following a stress caused by depletion of folate in colon cancer cells (43). Furthermore, in breast cancer, Hh signaling regulates the ability of stem like cells to generate tumor bulk (44).

The activation of this pathway is further associate with lipid metabolism. Indeed, defects in cholesterol biosynthesis results in Hh signaling arrest in embryonic development (44).

## Metabolic Profile of Gastric Cancer Stem Cells (GCSCs)

Gastric cancer stem cells (GCSCs) show a distinct expression of several surface markers. These include: CD44 (cluster of differentiation 44), EpCAM (epithelial cell adhesion molecule), LGR5 (leucine-rich, repeat-containing, G-protein–coupled receptor 5), ALDH1 (aldehyde dehydrogenase 1), CD133 (cluster of differentiation 133), and SOX2 (sex-determining region Y-box 2) (45–47). The positivity to these markers is associated with vascular and lymph node invasion, tumor size and response to chemotherapeutic drugs (48). Interestingly, CSCs metabolism has become an active field of innovative research to target cancer progression (32). Here, we reviewed the current knowledge about GCSCs metabolism and the therapeutic strategies that can be employed to target metabolic pathways.

### Glycolytic Metabolism

**Figure 2:**
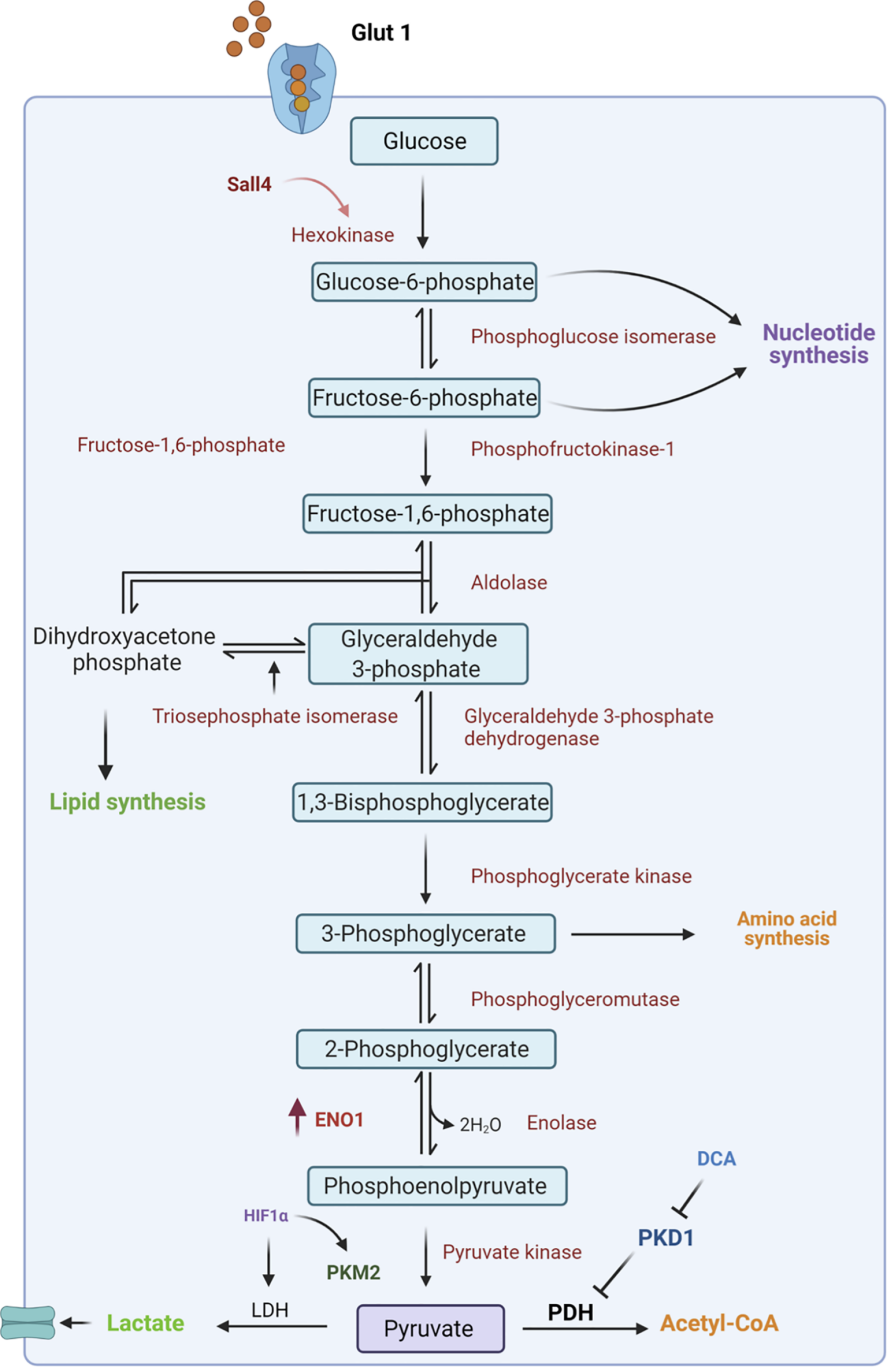
Schematic representation of glycolytic pathway. In CSCs, glucose from the extracellular environment is mainly metabolized through glycolysis. In particular, upregulation of several intermediates of this catabolic pathway, such as Hexokinase 2 and ENO1, leads to an increase in lactate production. Moreover, the inactivation of pyruvate dehydrogenase (PDH) by PDK1 is responsible for failure in the conversion of pyruvate to Acetyl-CoA and this results in increased lactate production. ENO1, Enolase1; HIF1α, hypoxia-inducible factor; LDH, Lactate Dehydrogenase; PKM2, Pyruvate kinase isozyme M2; PDH, Pyruvate dehydrogenase; PDK1, Pyruvate dehydrogenase kinase 1; DCA, dicloroacetate.

Glycolysis is the central pathway for glucose catabolism which can occur both in the presence of oxygen (aerobic) and in the absence of oxygen (anaerobic) (49, 50). In a hypoxic microenvironment, GCSCs reprogram their metabolism to adapt to lower oxygen levels through the upregulation of hypoxia-inducible factors (HIFs) (51). These factors regulate the expression of genes involved in glucose uptake and in the glycolytic pathway (52). Among these, GLUT transporters permit glucose entry through the plasma membrane allowing the cells to survive in low oxygen conditions (53, 54). Indeed, Yamada A. et al. reported that high Glut1 expression occurred at an early stage of gastric cancer where it facilitates 2-deoxy-2-[18F]fluoro-D-glucose ([18F]FDG) uptake (55), and it is responsible for gastric cancer progression by activating the AKT pathway (56), which plays a key role in glucose metabolism. However, the employment of specific inhibitors of Glut1 transporters, such as WZB117, has been demonstrated to affect tumor insurgence through the downregulation of stemness-associated genes, such as *SOX2, NANOG* and *BMI1* in different cancers including pancreatic, ovarian and glioblastoma cancer (57). This allows them to be utilized as a possible therapeutic strategy even in gastric cancer treatment. Subsequently to the higher intracellular glucose levels, Hexokinase 2 (HK-2), the first enzyme of the glycolytic pathway, is consistently overexpressed in gastric tumors and is associated with poor survival in patients with digestive system malignancies (58) (**Figure 2**). Recently, a novel molecular mechanism of metabolic regulation linking a key embryonic stem cells (ESCs) pluripotency factor SALL4 to HK-2 has been described in GC cells (59). Particularly, Shao et al. demonstrated that knockdown of SALL4 results in inhibition of glucose uptake and HK-2 activity. Conversely, SALL4 overexpression promotes cancer metabolic phenotype which can be reversed by HK-2 knockdown suggesting that this glycolytic enzyme is a downstream effector of the transcriptional factor SALL4. Indeed, targeting HK-2 in leukemic cells, with 3-bromopyruvate, leads to the dissociation of HK2 from mitochondrial membrane which is responsible for the enhanced sensitivity to the first-line chemotherapeutic drug (60). This approach could be used as a potential strategy to target GCSCs with high HK-2 activity.

Additionally, the overexpression of glycolytic enzyme Enolase 1 (ENO1) regulates stem cell-like characteristics of tumor cells and it is related to poor prognosis of GC (61). ENO1 overexpression promotes cisplatin resistance through a higher glycolytic activity. Moreover, the glycolytic phenotype can be modulated by Pyruvate kinase isozyme M2 (PKM2), an enzyme that has been proposed to be crucial for maintaining cell homeostasis. In GC, GCSCs, expressing CD44 surface marker, show an upregulation of PKM2 upon induction with CagA deriving from *H. Pylori* infection (62). It has been demonstrated that PKM2 overexpression plays a role in the stabilization of the transcriptional factor NF-κB that, through its binding to Bcl-xL anti-apoptotic protein, promotes GC development and progression (63). Inhibition of PKM2 achieved by shikonin hinders glycolysis in breast cancer cell lines making this compound a good candidate for other tumors (64).

The final step of glycolysis provides pyruvate molecules which can be converted in lactate through lactate dehydrogenase (LDH) or in Acetyl-CoA by pyruvate dehydrogenase (PDH) enzymes. The activity of PDH is negatively regulated by pyruvate dehydrogenase kinase 1 (PDK1) which is responsible for the interruption of the link between glycolysis and the TCA cycle, enhancing the conversion of pyruvate in lactate (65).

Alteration in the glucose metabolism by PDK1 activity is associated with a poor prognosis in gastric cancer. Targeting PDK1 with dichloroacetate (DCA) has been reported to induce metabolic changes that increase the cell sensitivity to chemotherapy (**Figure 2**) (66). As previously highlighted, although CSCs mostly utilize the glycolytic pathway, part of the energy is still produced by the OXPHOS pathway. To date, several drugs are available to reduce energy production *via* OXPHOS such as Metformin and Phenformin treatment (67, 68). These compounds inhibit the mitochondrial respiratory complex I delaying cancer growth *in vivo* and inducing apoptosis of CSCs (68). Furthermore, mitochondrial metabolism can be impaired by targeting mitochondrial protein biosynthesis and maturation. Indeed, treatments with tetracyclines, which hamper ribosomes, and Gamitrinib, an inhibitor of TRAP1 chaperone, result in OXPHOS reduction (69, 70). However, due to the metabolic plasticity of CSCs, dual inhibition of the glycolytic and OXPHOS pathways may represent a promising approach for CSCs targeting and tumor treatment.

### Lipid Metabolism

**Figure 3:**
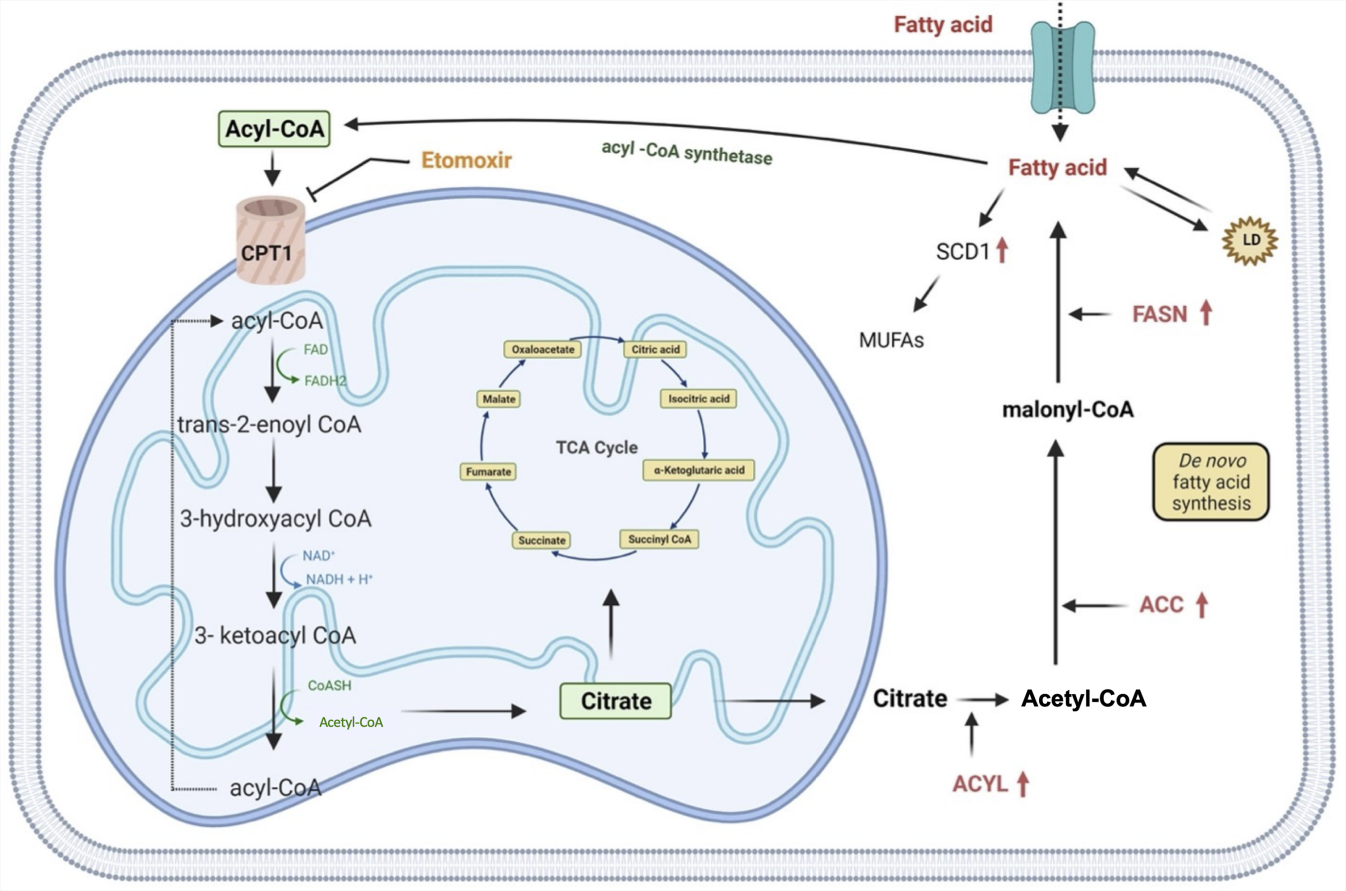
Representation of lipid metabolism. Rewiring of lipid metabolism including increased lipid uptake, lipid desaturation, *de novo* lipogenesis, lipolysis and FAO is necessary for supply GCSCs with energy. Extracellular FFAs are transported into cells and catabolized in mitochondria to produce acetyl-CoA which is converted in citrate and it enters in TCA cycle for the oxidation. Alternatively, *de novo* fatty acids synthesis begins with acetyl-CoA that is converted in malonyl-CoA and then in palmitate. Lipid catabolism can even occur through fatty acids esterification that allow them to be stored as triglycerides in lipid droplets. In addition, saturated fatty acids can also be converted into mono-unsaturated fatty acids by SCD1, an enzyme upregulated in gastric cancer. ACC, acetyl-CoA carboxylase; ACYL, ATP citrate lyase; FASN, fatty acid synthase; MUFAs, mono-unsaturated fatty acids; SCD1, stearoyl-CoA desaturase 1; CPT1, carnitine palmitoyltransferase 1; TCA, cycle, tricarboxylic acid cycle; LD, lipid droplet.

Lipids are essential components of the cell, both structurally and functionally, as they regulate the fluidity of the plasmatic membrane and they are involved in different cellular activities including cell-cell recognition, energy supplies and signaling transduction (71). Indeed, their metabolism is finely regulated by the cell through anabolic or catabolic pathways modulation, according to the availability of carbon source for *de novo* lipogenesis (44). Although an increase in lipid metabolism is already attributed to stem cells during somatic cell reprogramming (72), several reports describe the lipid metabolism rewiring of CSCs as a mechanism necessary to avoid death under unfavorable conditions (35). Recent studies show that exogenous absorption or endogenous synthesis of lipids plays a primary role in supporting CSCs’ self-renewal during the tumorigenesis process. Indeed, many of the enzymes responsible for lipid synthesis, such as ATP-citrate lyase (ACYL), acetyl-CoA carboxylase (ACC) and fatty acid synthase (FASN) are highly up-regulated in cancer (**Figure 3**) (44, 71). Likewise, inhibition of ATP-citrate lyase, the enzyme responsible for the conversion of cytosolic citrate to acetyl-CoA results in the downregulation of the transcriptional factor Snail, a key regulator of stemness phenotype in cancer stem cells (73). In particular, FASN, a key lipogenic enzyme that converts Acetyl-CoA and malonyl-CoA to palmitate, is drastically upregulated in many cancers such as breast, colon, lung, bladder, gastric, endometrial, ovary, kidney, pancreatic, head and neck, prostate, brain and melanoma (74–76). Inhibition of FASN activity with cerulenin induces regression in the formation of tumorspheres with a reduction in the expression of stemness markers, such as Nestin and CD133, and an increase in the expression of differentiation markers (77). Furthermore, comparative studies of metabolomic profiles between CSCs and non-tumor counterparts have shown that CSCs support their stemness by synthesizing a greater amount of monounsaturated lipids (MUFAs) (78). Lipid desaturation, or the conversion of saturated lipids into unsaturated lipids, is mediated by specific enzymes, such as stearoyl-CoA desaturase-1 (SCD1). In gastric cancer, SCD1 has found to be upregulated and this results in poor survival rates. Furthermore, this enzyme is able to induce tumorigenesis, drug resistance and metastasis by regulating GCSCs proliferation through the Hippo pathway (79). Hence CSCs, compared to their differentiated tumor counterparts, show an enhancement of lipid synthesis *de novo*, which is also reflected in the accumulation of intracellular lipids. An important role of lipid droplets (LD) located at the cytoplasmic level in supporting the excessive metabolic demand of CSCs has also been advanced. LD, a source of accumulation of fatty acids in the form of triglycerides, act as a reservoir of energy for the sustenance of CSCs in glucose deprivation condition (80). Moreover, another strategy that CSCs activate in a low glucose condition is represented by the β-oxidation of free fatty acids (44). Thus, Nanog, a stem cell marker, reprograms the metabolism of tumor-initiating stem-like cells (TIC) by repressing the expression of OXPHOS genes and activating the fatty acids oxidation (FAO). This allows tumor-initiating stem-like cells to maintain self-renewal and drug resistance (81). It has been demonstrated that, in GC, mesenchymal stem cells (MSCs) promote the synthesis of MACC1-ASI, a lncRNA that is responsible for FAO-dependent stemness and chemo-resistance insurgence. Furthermore, it has been found that the downregulation of CPT1, the FAO rate-limiting enzyme, reduced stemness and resistance to 5-FU and oxaliplatin. Accordingly, inhibition of CPT1 with Etomoxir allows reversing the resistance that mesenchymal stem cells show to the FOLFOX therapeutic regimen (**Figure 3** and **Table 1**) (82). However, a recent report describes a lipid metabolism shift that occurs when stem cells switch from a quiescent state to a proliferative state (88). This represents a paradox because, in a quiescent condition, cells rely on β-oxidation to produce energy for the cell viability and stemness phenotype maintenance as the citrate is first converted to succinate and, then, to malate. The selective inhibition of this catabolic pathway resulted in a loss of the quiescence state and in the acquisition of a differentiated phenotype (88).

**Table 1:** List of compounds targeting CSCs metabolism.

Compound	Target	Tested Tumor Type	Reference
WZB117	Glut-1	PANC-1 CSLC (pancreatic cancer) A2780 CSC (ovarian cancer) GS-Y03 (glioblastoma)	(57)
3-bromopyruvate	HK2	K-562 (leukemic cells)	(60)
Shikonin	PKM2	MCF-7, MCF-7/Adr, MCF-7/Bcl-2, MCF-7/Bcl-x(L) and A549 (breast cancer)	(64)
Dichloroacetate (DCA)	PDK1	MKN45, AGS (gastric cancer)	(66)
Metformin/Phenformin	Mitochondrial Respiratory Complex I	Multiple Tumor Cell Types	(67, 68)
Gamitrinib	TRAP1	PC3 (prostate cancer)	(69)
Tetracyclines	Ribosomal Subunit 30S	Multiple Tumor Cell Types	(70)
Cerulein	FASN	G144, Y10 (glioma)	(77)
Etomoxir	CPT1	MKN45, AGS (gastric cancer)	(82)
BPTES	GLS	P493 (B cell lymphoma)	(83)
R162	GDH1	Multiple Tumor Cell Types	(84)
Benzilserine	ASCT2	HGC-27, NUGC-3, MKN45, MGC-803, AGS, MKN74 (gastric cancer)	(85)
Mitoketoscins	Unknown	MCF7 (breast cancer)	(86)
Sulfasalazine	xCT	MKN28 (gastric cancer)	(87)

HK-2, Hexokinase 2; PKM2, Pyruvate kinase isozyme M2; PDK1, Pyruvate dehydrogenase; FASN, fatty acid synthase; CPT1, carnitine palmitoyltransferase 1; GLS, glutaminase; BPTES, bis-2-(5-phenylacetamido-1,3,4-thiadiazol-2-yl) ethyl sulfide ASCT2, Alanine/Serine/Cysteine-preferring Transporter 2; GDH, glutamate dehydrogenase 1.

Along with the metabolism of fatty acids, cholesterol metabolism is also a hallmark of cancer. In particular, an increase in the levels of SREBP2, a transcription factor responsible for cholesterol biosynthesis and homeostasis, is correlated with an increase in the tumor stem cell bulk population (89). *De novo* lipidic synthesis and the oxidation of fatty acids represent the new metabolic targets to inhibit CSCs self-renewal and identifying new inhibitors of the key players involved could slow down the growth of the tumor mass (90).

### Exploring the Role of Glutamine Metabolism

**Figure 4:**
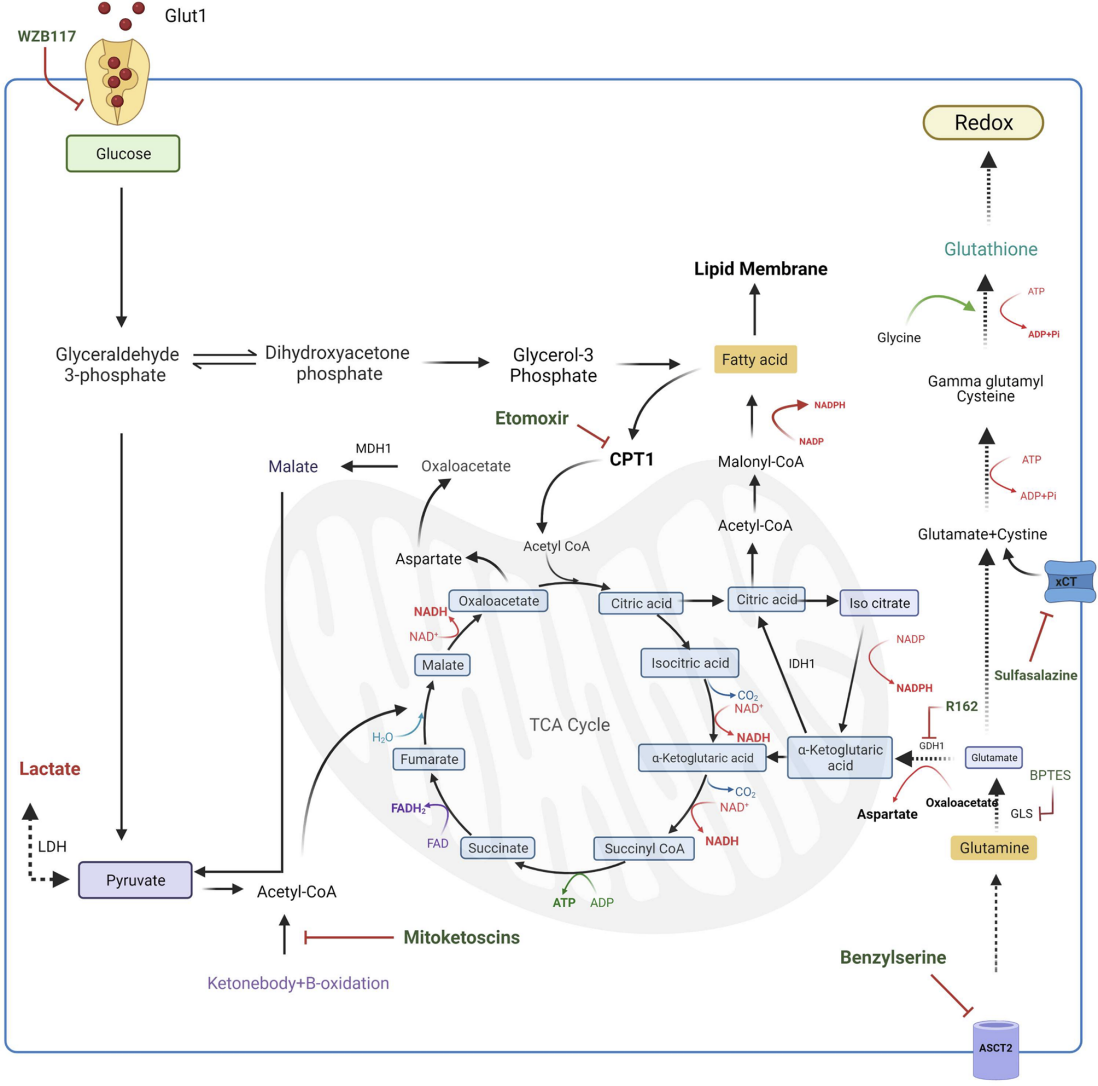
Targeting of metabolic pathways in CSCs. Several compounds inhibit different metabolic intermediates in CSCs. Glut1 uptake can be inhibited by WZB11 treatment resulting in a lower energy production. Also targeting the glutamine metabolism represents a potential strategy to apply in cancer treatment. Indeed, inhibition of ASCT2 transporter with Benzylserine prevents the glutamine uptake while blocking the metabolic intermediates, such as GLS with BTES, hinders glutaminolysis. Moreover, glutamate deriving from processed glutamine, together with intracellular cysteine, can participate in glutathione synthesis that is responsible for low ROS maintenance. Another strategy to increase CSCs death is the reduction of cysteine uptake by inhibition of xCT transporter with Sulfasalazine. CSCs survival can be also hampered by targeting the conversion of ketonebodies into Acetyl-CoA, a substrate necessary for TCA cycle. Moreover, also lipid entering in TCA cycle can be inhibited using selective inhibitors of enzymes involved in lipid metabolism. Indeed, treatment of CSCs with Etomoxir, results in the CPT1 enzyme inactivation resulting in the cytosolic accumulation of lipidic intermediates. TCA, tricarboxylic acid cycle; G6PD, Glucose-6-phosphate dehydrogenase; LDH, lactate dehydrogenase; GLS, glutaminase; GDH1, glutamate dehydrogenase 1; ASCT2, Alanine/Serine/Cysteine-preferring Transporter 2; IDH1, isocitrate dehydrogenase; xCT, cysteine/glutamate transporter; CPT1, carnitine palmitoyltransferase 1; MDH1, malate dehydrogenase; BPTES, bis-2-(5-phenylacetamido-1,3,4-thiadiazol-2-yl) ethyl sulfide.

Glutamine metabolism is an essential mechanism that CSCs display to produce macromolecules, such as lipids, proteins and nucleic acids, that are indispensable for proliferation (91). To date, no evidence describes the role of this metabolism in gastric cancer stem cells, however, given its importance as the carbon source in other cancer stem cells, further investigation should be carried out to understand the metabolic function of glutamine in gastric stemness. In particular, in cancer stem cells glutamine is used as a pivotal source of nitrogenous as it represents a good donor of reduced nitrogen for building both purine and pyrimidine bases as well as proteins (91). Furthermore, glutamine acts as a carbon source for the TCA cycle through its conversion to alpha-ketoglutarate (α-KG) (92). Specifically, glutamine is first converted to N-acetyl-glucosamine by glutaminase (GLS), a MYC regulated enzyme, then converted to α-KG by glutamate dehydrogenase (GDH) and then it enters in TCA cycle for energy production. This mechanism is defined as oxidative glutaminolysis (93). Targeting these enzymes could represent a good pharmacological strategy for potential therapy in different types of cancer. Indeed, the use of a specific inhibitor targeting GLS leads to an inhibition of MYC-induced B-cell lymphoma and MYC-induced hepatocarcinoma (83), while, GDH inhibition results in lower α-KG levels and high ROS production, resulting in hindering of cancer cell proliferation and tumor progression (94). Thus, this emerging evidence shows that increasing glutamine metabolism promotes tumor growth despite the regulation of redox homeostasis (84). However, beyond the oxidative glutaminolysis, glutamine can follow a reductive carboxylation pathway. Indeed, in a hypoxic microenvironment, glutamine can also be converted to citrate by the reductive activity of NADP^+^-dependent isocitrate dehydrogenase 1 (IDH1) leading to Acetyl-CoA production that is used for fatty acid synthesis and it is necessary to guarantee substrates for cell proliferation (91). This makes glutamine metabolism a crucial point for the regulation of survival, proliferation and differentiation of cancer stem cells. Recent studies describe glutamine metabolism as a possible target in cancer therapy. Several compounds are described being able to modulate different intermediates of this metabolic pathway. The first-line strategy to regulate glutamine metabolism could be the inhibition of glutamine transporter ASCT2 with benzylserine to prevent the uptake of this amino acid and its employment in anabolic and catabolic pathways (85). Subsequently, intracellular glutamine is converted into glutamate through glutaminase (GLS) activity; the inhibition of GLS with bis-2-(5-phenylacetamido-1,3,4-thiadiazol-2-yl) ethyl sulfide (BPTES) is associated with DNA replication arrest leading to cell death in the B lymphoma cell line (83). Another targetable step is represented by the conversion of glutamate into α -ketoglutarate acid through the activity of glutamate dehydrogenase 1 (GDH1). Targeting GDH1 with R162 prevents glutamine entry in the TCA cycle increasing ROS levels in several cancer cell lines (84). Also, ketone-bodies are used as a substrate for energy production in cancer stem cells as they are converted in Acetyl-CoA which enters in the TCA cycle providing carbons for energy production. A new class of compound, named “mitoketoscins”, has been found to impair this conversion leading to the inhibition of breast cancer stem cells proliferation. However, the molecular mechanism of mitoketoscins still remains unclear (**Figure 4**) (86).

## Regulation of Redox State in GCSCs

GCSCs like normal stem cells, exhibit a tightly regulated metabolism that governs their function. It is well known that, in stem cells, intracellular levels of Reactive Oxygen Species (ROS) play a pivotal role in the regulation of the balance between self-renewal and differentiation (95, 96). Indeed, while mitochondrial ROS production has been implied to have a role in muscle and adipocyte differentiation (97, 98), low levels of ROS are actively maintained in stem cells *via* aerobic glycolysis (99), and high activity of antioxidant machinery. Also CSCs, like normal stem cells, display strictly regulated ROS production through the metabolic switch and upregulation of the radical scavenging system including the members of the superoxide dismutase family (SODs) or glutathione peroxidase family (GPXs) (100–102). Furthermore, it has been demonstrated that increased ROS levels may enhance the cell sensibility to drugs, reducing chemo-resistance, while, on the other hand, low intracellular ROS levels may have a protective function in tumor bulk (103, 104). A recent study demonstrated that CD13, a CSCs marker, negatively regulates ROS levels, resulting in increased stemness in liver CSCs (105). Further, in breast cancer, CD24^-/low^/CD44+ initiating cells show low levels of radiation-induced ROS that confer higher tumorigenicity and resistance to radiation (106). This evidence endorses the association between low levels of intracellular ROS and cancer stemness even if, as for normal stem cells, the redox status of CSCs is not well-defined. Glutathione (GSH) is an antioxidant peptide with a high abundance in mitochondria of eukaryotic cells. GSH is involved in the maintenance of redox balance through ROS detoxification, and in the protection of phospholipids in the mitochondrial membrane (107). Interestingly, high glutathione levels are found in embryonic stem cells (108) and mesenchymal stem cells where they are responsible for the maintenance of stemness (109). Likewise, high GSH levels together with GSH-related enzymes are found in CSCs from gastric (110, 111), liver (105) and breast cancer (112). Conversely, little is known about the glutathione pathway in CSCs. It has been demonstrated that pancreatic CSCs show high levels of GSH content and upregulation of several genes involved in the GSH signaling (111). As for CSCs of other carcinomas, stem cells from gastric cancer, marked by upregulation of a variant of CD44 receptor (CD44v) (113, 114), display an enhanced capacity of glutathione synthesis and defense against ROS. In particular, high levels of intracellular GSH are due to the high activity of the plasma membrane transporter xCT, a subunit of the cystine–glutamate exchange transporter which is involved in the cysteine uptake, essential for GSH synthesis (110, 115). It is well established that cancer stem cells activate the p38 MAPK pathway to face oxidative stress (116). In particular, ROS production induces ASK1 kinase activation, which, through MAPK3/4/6 activation (117) leads to phosphorylation and activation of p38. Phosphorylated p38 is responsible for apoptosis activation and growth arrest, having so, a negative role on tumorigenesis (118). In GCSCs, the interaction of CD44v with xCT leads to an increased intracellular GSH which results in ROS-p38 MAPK suppression and enhanced tumor development (110, 115). Then, a specific therapy targeted to the CD44v-xCT pathway may impair the ability of GCSCs to protect themselves from oxidative stress, increasing the sensitivity to cancer available treatments. Indeed, inhibition of xCT transporter by sulfasalazine sensitizes gastric cancer stem cells to the drugs with a positive effect on the clinical efficacy of chemotherapy (**Figure 4** and **Table 1**) (87).

## Role of miRNAs in the Metabolic Reprogramming of GCSCs

MiRNAs are small single-stranded RNAs capable of binding to the 3’UTR of mRNAs inducing inhibitory signals to the ribosome which detaches from the mRNA blocking the translation of the protein (119). It is already known that these small non-coding RNAs are able to regulate the expression of many genes involved in cellular homeostasis such as progression, cell cycle, migration, apoptosis and cell differentiation (120). In cancer cells, the expression of miRNAs is deregulated through genetic and epigenetic modifications. Indeed, it has been found that the overexpression of some miRNAs (oncomiR), due to genomic amplification of their coding region, negatively regulates the levels of tumor suppressor genes. Conversely, deletions or loss of function mutations in miRNAs coding regions, which regulate proto-oncogenes, leads to reduced control over cell growth and differentiation, unlocking their tumorigenic potential (121). The analysis of stem-like gastro-spheres highlights deregulation of several miRNAs such as miR‐21, miR‐10b, and miR‐146a which are responsible for upregulation stem cell-related genes, clonogenicity ability and chemotherapeutic resistance (122–124). Indeed, the comparison between GCSCs and their non-stem cancer cell counterparts, through RT-PCR analysis, has shown a differential expression in miRNA, suggesting that the pathways governing these two cell populations are different (125).

Little is known about the role of miRNAs in the metabolic reprogramming of GCSCs. In CSCs, as already discussed, the glycolytic pathway plays a pivotal role in energy production and several miRNAs are described to be involved in this process. HK-2, the first rate-limiting enzyme of glycolysis, can be modulated by miR-181b which has a binding site in the 3′-untranslated region of HK-2 transcript and found to be down-regulated in gastric cancer tissues (126). Likewise, a comparison between gastric cancer tissues and adjacent noncancerous gastric mucosa tissues revealed the down-regulation of miR-422a expression level which inversely correlates with tumor size and depth of infiltration. He et al. pointed out how miR-422a is linked to metabolism, since it represses PDK2 activity, restoring pyruvate conversion in Acetyl-CoA through the PDH enzyme (127). A different role is attributed to miR-200 which appears to have conflicting effects in promoting stemness associated with oxidative stress (128). Also in GC were found non-canonical miRNAs that are independent from Drosha, an enzyme responsible for miRNA processing. In particular, it has been found that miR-6778-5p maintains the stem properties of GCSCs by blocking the YWHAE/c-MYC axis responsible for the reduced expression of SHMT1, a cytosolic isoenzyme involved in the metabolism of one-carbon folate-dependent. Thus, an increase in the expression of SHMT1 dependent on miR-6778-5p promotes the maintenance of stemness and enrichment of GCSCs (129). Furthermore, several studies show that lncRNAs can be involved in the post-transcriptional regulation process by interacting with miRNAs (130). As already mentioned, in GC, lnc-MSCC1-AS1 promotes stemness and chemo-resistance through the reprogramming of lipid metabolism. This role has been attributed to antagonism with miR-145-5p which promotes apoptosis of tumor cells through an increase in ROS levels and drug-associated toxicity (82). Thus, given the growing importance that has been attributed to miRNAs, it is necessary to improve the current knowledge of these biomolecules and their involvement in the metabolic reprogramming processes.

## Conclusion

Over the course of this decade, substantial evidence suggested that CSCs are accountable for the progression of various types of cancer, including GC. These cells have been shown to have a distinct metabolic phenotype that depends on the microenvironment and genetic background. Several reports suggest that the metabolic rewiring is responsible for the conventional chemotherapy and molecularly targeted therapy failure in GC patients, resulting in poor clinical outcomes. As described, the OXPHOS and glycolysis are the primary sources of energy even if several regulatory pathways participate in this metabolic cycle for CSCs. Numerous factors are involved in the regulation of metabolic shifting, and for this reason, it is challenging to establish a targeted cancer therapy. Therefore, revealing the molecular mechanisms accountable for genetic mutation and epigenetic changes in GCSCs or the discovery of new metabolic targets that regulate tumor development and drug resistance might be a possible therapeutic opening for GC. However, focusing on alternative approaches should be the best way for patients’ benefits. These approaches include: to find out the specific CSCs markers to establish an early diagnosis of GC; to define the metabolic profile of tumor-inducing CSCs; to target the metabolic pathways, which play a crucial role in triggering the transitional states of normal to gastric cancer stem cells. We are just at the beginning of understanding metabolic reprogramming from normal stem cells to GCSCs. More studies should be done to increase the knowledge about this mechanism in order to improve the quality of life of GC patients.

## Author Contributions 

Conceptualization, PM and GF. Writing—original draft preparation, MA, GDP, HV and SL. Writing—review and editing, SR, PZ and PM. Supervision, GF and PM. All authors contributed to the article and approved the submitted version.

## Funding

This research was supported by PRIN 2017 (ENTI DI RICERCA DI RILEVANTE INTERESSE NAZIONALE) -Prot.2017XJ38A4.

## Conflict of Interest

The authors declare that the research was conducted in the absence of any commercial or financial relationships that could be construed as a potential conflict of interest.
